# A Novel Remote Sensing Image Registration Algorithm Based on Feature Using ProbNet-RANSAC

**DOI:** 10.3390/s22134791

**Published:** 2022-06-24

**Authors:** Yunyun Dong, Chenbin Liang, Changjun Zhao

**Affiliations:** 1 Northwest Land and Resource Research Center, Shaanxi Normal University, Xi’an 710062, China; zhaocj@snnu.edu.cn; 2 State Key Laboratory of Management and Control for Complex Systems, Institute of Automation, Chinese Academy of Sciences, Beijing 100190, China; liangchenbin2019@ia.ac.cn; 3 School of Artificial Intelligence, University of Chinese Academy of Sciences, Beijing 100190, China

**Keywords:** image registration, feature matching, RANSAC, probability-guided RANSAC

## Abstract

Image registration based on feature is a commonly used approach due to its robustness in complex geometric deformation and larger gray difference. However, in practical application, due to the effect of various noises, occlusions, shadows, gray differences, and even changes of image contents, the corresponding feature point set may be contaminated, which may degrade the accuracy of the transformation model estimate based on Random Sample Consensus (RANSAC). In this work, we proposed a semi-automated method to create the image registration training data, which greatly reduced the workload of labeling and made it possible to train a deep neural network. In addition, for the model estimation based on RANSAC, we determined the process according to a probabilistic perspective and presented a formulation of RANSAC with the learned guidance of hypothesis sampling. At the same time, a deep convolutional neural network of ProbNet was built to generate a sampling probability of corresponding feature points, which were then used to guide the sampling of a minimum set of RANSAC to acquire a more accurate estimation model. To illustrate the effectiveness and advantages of the proposed method, qualitative and quantitative experiments are conducted. In the qualitative experiment, the effectiveness of the proposed method was illustrated by a checkerboard visualization of image pairs before and after being registered by the proposed method. In the quantitative experiment, other three representative and popular methods of vanilla RANSAC, LMeds-RANSAC, and ProSAC-RANSAC were compared, and seven different measures were introduced to comprehensively evaluate the performance of the proposed method. The quantitative experimental result showed that the proposed method had better performance than the other methods. Furthermore, with the integration of the model estimation of the image registration into the deep-learning framework, it was possible to jointly optimize all the processes of image registration via end-to-end learning to further improve the accuracy of image registration.

## 1. Introduction

With the explosive growth of remote sensing images, the integrated application of multi-source images with varied resolutions and at different times have become a focus of research in order to maximize their value [1], including methods such as image fusion, change detection, image mosaic, long-term series analysis, and so on. However, image registration is a prerequisite for a broader and deeper application of remote sensing images, and the accuracy impacts the effectiveness and even the success in subsequent applications [2]. Therefore, accurate image registration is an indispensable preprocessing procedure for remote sensing application [3,4].

In general, image registration methods have been roughly divided into two categories: Area-based methods and feature-based methods [5,6]. Area-based methods consider the intensity of images, and they focus on how to define a similarity measurement, such as correlation-type [7] and mutual information methods [8]. The advantage of these approaches is that the salient structures are not required in the image, and the disadvantage is that they are vulnerable to non-translation geometric deformation and non-linear radiation distortions. Therefore, these approaches are usually applied after the coarse registration of remote sensing images or to images with minor gray differences [9,10].

However, feature-based image registration methods analyze arbitrary, complex geometric deformations only if salient structures exist in the images. Therefore, the feature-based image registration approaches have good flexibility and utility in practice. In the process of image registration based on features, there are four main steps including feature extraction, feature descriptor, feature matching, and transformation model estimation [5,6].

Feature extraction. For images to be registered, some sparse features across each image are extracted. The feature extraction algorithms include gradient, intensity, contour-curvature, and convolutional neural-network-based approaches [11]. The extracted features are low-level features, such as point features (corner, cross point of lines, etc.), line features (straight line, edges, ridges, etc.), and polygon features (lake, buildings, etc.) from the image. The commonly used measurements for features in image registration include repeatability, distinctiveness, accuracy, and quantity [12]. For line and polygon features, the evident line or polygon structures are required in the image. Therefore, in practical applications, the typically used features are corner points, and the representative feature extraction operation is difference of gaussian (DoG) [13], which can extract keypoints with good performance in terms of position, repeatability, rotation invariance, and scale invariance. Subsequently, a series of improvements have been proposed. They first inherit the property from the scale pyramid of DoG and then make an improvement in some specific aspect. For example, affine-SIFT (ASIFT) [14] increases the affine invariance by simulating all image views and SURF [15], which improves the computation efficiency by relying on integral images. Recently, with the excellent performance of deep-learning-based methods in the tasks of computer vision, feature extraction based on deep learning has been proposed. Key.Net [16], which can detect robust keypoints using a combination of handcrafted detectors and learned convolution neural networks (CNNs), introduced multi-scale loss and the ranking of stability of keypoints across scales. This approach mimics the behavior of handcrafted detectors by stacking convolution filters of different sizes. However, for remote sensing image registration, the low volume of training images publicly available has limited its application.

Feature description. Once a discriminating feature is extracted, a local patch centered on the feature point is used to generate a descriptor to determine the corresponding feature from other images. According to the information gained from the local patch, the feature descriptor can be roughly grouped by either gradient-, intensity-, or binary-based methods. The core measurements for the feature descriptor are its robustness to geometrical variety, gray changes, and discrimination [12]. As compared to the binary- and intensity-based methods, the gradient-based method has good discrimination ability and robustness, but at the expense of computational complexity. The representative descriptor is a 128-dimension SIFT descriptor that is generated by dividing the local patch into 4×4 non-overlapping cells and conducting a histogram of gradient orientations with 8 bins for each cell. Namely, the strategies of pooling and statistics ensure that the SIFT descriptor is robust to geometrical transformation. In addition, the raw patch centered on a feature point can be fed into a CNN to generate a descriptor as a representation for the input patch [17,18]. Due to the strong ability of information representation in CNNs, the descriptor generated by a CNN always has efficient performance. However, the generality of the descriptor generated by CNNs for different datasets has limited its widespread application, especially when a large volume of remote sensing training images is not available.

Feature matching. Each extracted feature has a corresponding feature descriptor, which is designed to identify the feature. Generally, the feature matching consists of two stages: putative matching and final matching, namely the removal of false matches from the putative matching sets. In the stage of the putative matching, correspondence is established between extracted features from the reference and sensed images, according to the feature descriptor similarity, namely the distance in the measuring space. The commonly used strategies are predefined threshold (PT), nearest neighbor (NN), mutual nearest neighbor (mNN), and nearest neighbor–distance ratio (NNDR). The PT strategy recognizes the matches with a distance below a predefined threshold. This strategy is sensitive to image and may incur one-to-many matchings, which violates the nature of one-to-one correspondence. The NN strategy can alleviate the problem of data sensitivity but may still result in a one-to-many result. For the mNN strategy, each feature in the sensed image identifies its NN feature (and vice versa), the feature pairs that are mutual NNs become candidate matches. It can solve the one-to-many problem, but at the expense of filtering out many other potential true matches. The NNDR strategy, by calculating the distance ratio of the first and second nearest neighbor, recognizes the candidate matching with the NNDR greater than the predefined threshold. In practical application, it can obtain efficient performance while not filtering out many true matches by mistake. Therefore, the NNDR strategy is broadly used in SIFT descriptor matching.

Transformation model estimation. Compared to the natural images, due to the characteristic of satellite imaging, attitude is relatively stable, and the attitude parameters are typically accurate. The geometrical deformations between remote sensing images are relatively simple, and the projective transformation, homography transformation, or even the affine transformation can align them [19]. Therefore, in the process of transformation modeling, it is easy to implement and finish the remote sensing image registration.

In the aforementioned four stages of image registration based on feature, each stage is indispensable and has an impact on the final performance of image registration. Among them, feature matching has attracted increasing attention, as it is the cornerstone of accurate model estimation. However, due to the effect of various noises, putative feature matching is often noisy, and further refining processes are necessary for robust and accurate model estimation. For the refining process of putative feature matching, the global or local geometrical constraints are introduced to remove the false matches in the putative matching sets. The most representative method of mismatch removal is random sample consensus (RANSAC) [20]. It follows the paradigm of hypothesize-and-verify: sample a minimum subset from the subset data, as required for a model; estimate the definite model as the hypothesis; and verify the quality of the estimated model by a given measure. The process is repeated until a termination condition is reached and then returns the best model identified. However, when the ratio of outliers of putative feature matching increases, the efficiency of RANSAC drops dramatically as it requires exponentially many iterations to identify an outlier-free minimum set. In practice, considering the balance between accuracy and efficiency, the maximum iterations are usually the termination criteria, which results in failing to identify the best model, even a wrong model. To identify a good model, improvements have been proposed in two aspects: efficiency and the quality of the final estimate. In terms of efficiency, researchers have focused on the sampling of a minimum outlier-free set and the rapid verification of the model. For example, PROSAC [21] uses side information to change the order in which RANSAC samples minimum sets; whereas GroupSAC [22] draws the minimum sets from fewer groups by using a hierarchical sampling paradigm, and the sequential probability ratio test (SPRT) [23] was adopted to accelerate the model verification of RANSAC. In terms of the quality of the final estimated model, due to minimum subsets amplifying the effect of noise and returning a hypothesis that is inaccurate, locally optimized RANSAC [24] is conducted when the *so-far-the-best* model is obtained. These promising improvements have been integrated into a universal RANSAC (USAC) framework [25]. However, that has been no explicit or efficient method for obtaining the additional information regarding the corresponding feature matching, so the above-mentioned variants of RANSAC have not been effective.

Motivated by the superior performance of deep learning in many computer vision applications and the works of differential RANSAC [26,27], where the deterministic hypothesis selection of RANSAC was replaced by a probabilistic selection, we designed a deep convolutional neural network to predict the quality of corresponding feature points in a putative matching set in the form of probability. Then we utilized the probability generated by the deep convolutional neural network to guide the minimum set sampling of RANSAC, which we referred to as ProbNet-RANSAC. We then incorporated ProbNet-RANSAC into the image registration pipeline in an end-to-end learning approach. Together with the remote sensing training images we created in a semi-automated process and task-loss function, we trained ProbNet to predict the probability of the outlier-free correspondence points in the putative matching sets. With the help of ProbNet-RANSAC, we efficiently acquired a minimum-correct correspondence point set and accurately estimated the transformation model to improve the image registration accuracy. In summary, our contributions were as follows:

- A semi-automatic method of creating training data for remote sensing image registration was proposed that could greatly reduce the workload of labeling and make it possible to train a deep convolutional neural network.
- We derived the model estimation based on RANSAC from a probability perspective, and a deep convolutional neural network of ProbNet was built to evaluate the quality of corresponding feature points according to probability.
- We used the probability generated by ProbNet to guide the sampling of a minimum set of RANSAC, which could acquire a more accurate transformation model.

## 2. Methodology

In this section, we describe the image registration pipeline based on feature by using ProbNet-RANSAC in detail and focus on the process of the geometric transformation model estimation based on ProbNet-RANSAC.

### 2.1. The Whole Workflow of Image Registration Based on Feature by Using ProbNet-Ransac

**Figure 1:**
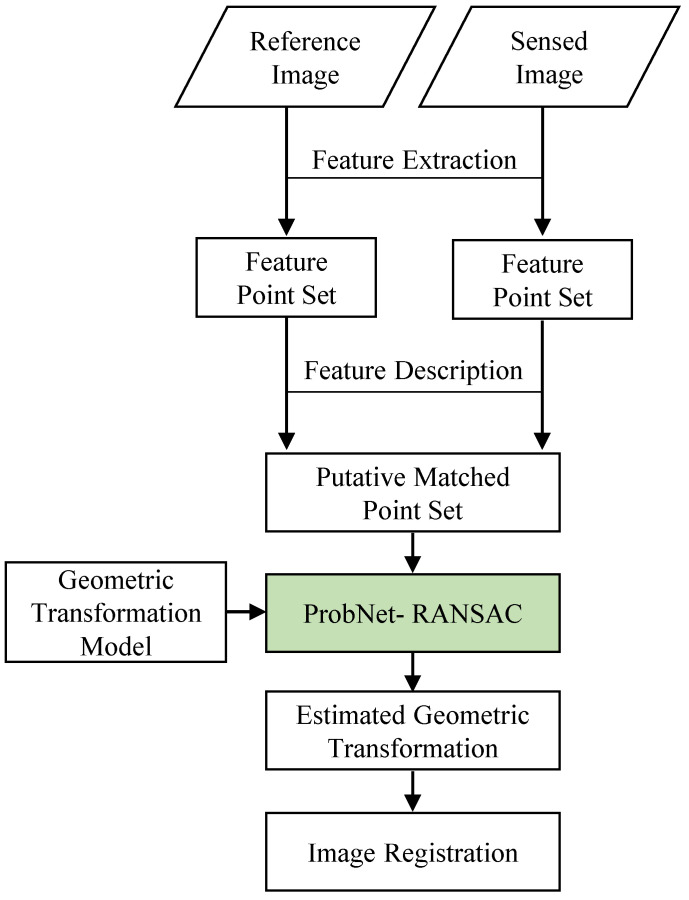
The flowchart of image registration based on feature by using ProbNet-RANSAC.

Here, for the simplicity of description, we take the registration of a pair of remote sensing images as an example. The flowchart of image registration based on feature by using ProbNet-RANSAC is shown in Figure 1.

There are four steps in the process. (1) Feature extraction. For each image, we utilized the feature extractor to extract the salient points and obtain two feature point sets; (2) Feature description. For each feature point in these two feature point sets, a corresponding feature descriptor was generated. According to a similarity measure and general filter rules, a putative corresponding point set was acquired; (3) Transformation model estimation. According to the prior knowledge of geometric distortion between images, we determined the category of the geometric transformation model, such as affine transformation, similarity transformation, and so on. Coupling that with the geometric transformation model, ProbNet-RANSAC used the putative corresponding point sets as input to efficiently and accurately estimate the geometric transformation. This was to our main contribution, and it is detailed in the following section; (4) Image registration. Once the estimated geometric transformation was acquired, we accomplished the image registration by resampling the sensed image based on the estimated geometric transformation.

### 2.2. The Pipeline of Geometric Transformation Model Estimation Based on ProbNet-Ransac

**Figure 2:**
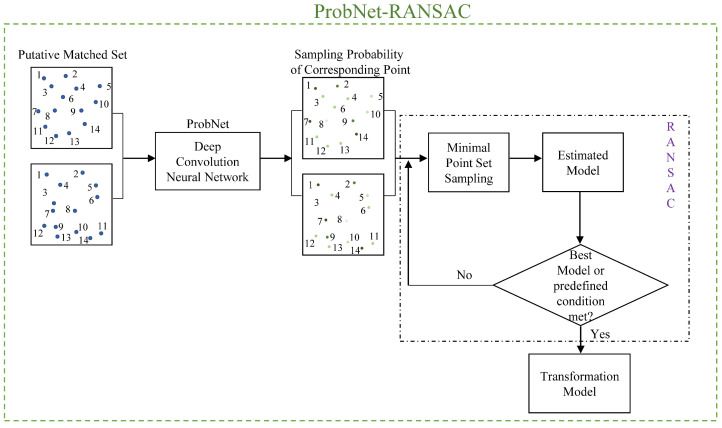
The schema of geometric transformation model estimation based on ProbNet-RANSAC.

After processing of the feature extraction and feature description, each feature point had its own feature descriptor. In general, the feature descriptor’s Euclidean distance was adopted as a measurement, and the popular strategy of nearest neighbor distance ratio (NNDR) [13] was applied to acquire the putative matched point set. Therefore, the acquired putative matched point set was taken as the input for the geometric transformation model estimation. The schema of geometric transformation model estimation based on ProbNet-RANSAC is shown in Figure 2.

We fed all the putative matched point pairs acquired from a pair of images into a deep convolutional neural network to generate a probability for each pair of matched points; the deep convolutional neural network was referred to as ProbNet. The probability of each matched points pair indicated its likelihood to follow the geometric transformation model. The architecture of ProbNet and the optimization of ProbNet is detailed in the following sections. Under the guidance of the generated probability of each matched points pair, we drew the minimum matched point set and estimated the geometric transformation model based on the sampled minimum point set by following the schema of RANSAC. We repeated the process until the best model was found or the predetermined iteration termination condition was met. Once the iteration process terminated, we chose the best geometric transformation model and regarded it as the final transformation model.

#### 2.2.1. The Architecture of Deep Convolution Neural Network (ProbNet)

**Figure 3:**
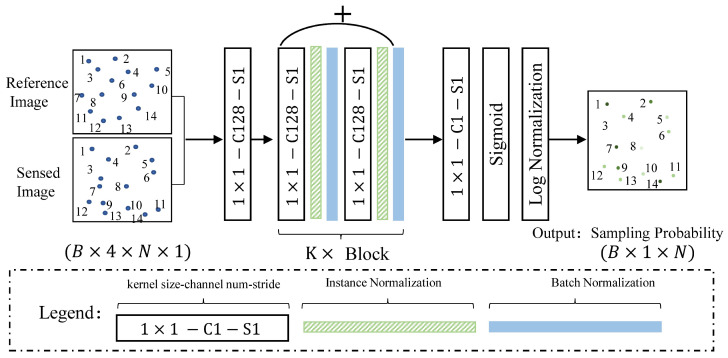
The architecture of ProbNet for generating the sampling probability. It consisted of three core components. 1. A convolutional layer with kernel size of 1×1, resulting io a fully connected layer that is firstly utilized to transform the matched points coordinates into a higher dimension space. 2. Stacked K residual blocks. Each residual block consisted of 1×1 convolution layers interleaved with instance normalization and batch normalization layers. The arc with a symbol of plus denotes a skip connection for each block. 3. The probability generation layers consisted of one 1×1 convolutional layer, which reduces the dimension of output of the stacked residual blocks to 1, and then uses sigmoid and Log normalization layers to generate the final sampling probability.

For ProbNet, its input was the aforementioned matched points in the putative matched point set, and its output was a log probability for each matched point, which indicated that the log possibility of the input matched points following the geometric transformation model. Motivated by the work of [27] and the function of commonly used convolution components of fully connected layer, residual block, and instance norm layer, we built ProbNet, whose architecture is shown in Figure 3.

To illustrate the architecture of ProbNet more clearly, we supposed that there were *N* pairs of matched points in the putative matched point set. First, we concatenated the coordinates of the matched points into a 4×1 coordinate vector, and we obtained N×4×1 matrix data by concatenating all the *N* coordinate vectors. Assuming that there were *B* pairs of images to be processed by ProbNet during the training, we could then obtain the input data with a shape of B×N×4×1. We fed the input data into the network of ProbNet and finally obtained a resulting matrix with shape of B×1×N vector, namely B vectors with shape of 1×N, where each element denoted the log probability of sampling its own corresponding matched points, and the sum-exp of all the *N* elements was equal to 1. The architecture consisted of convolutional layers with kernel sizes of 1×1, residual blocks, and instance normalization layers. The 1×1 convolutional layer was used as feature transformation and transformed the original coordinate vector into a higher dimension feature space, and ensured that the order of matched points was independent of the result of the feature transformation at the same time. Each residual block was composed of two 1×1 convolution layers interleaved with instance normalization layer and batch normalization layer. The instance normalization is used to infuse global context, which encodes the scene geometry. The batch normalization layer is used to accelerate training. Then the same *K* residual blocks are connected by the skip connection, which constitute the main body of ProbNet. Finally, the output of *K* residual blocks is processed by a linear convolutional layer to reduce its feature dimension to 1, and a sigmoid layer and Log Normalization layer were utilized to process the results further to obtain the log probability, which was beneficial to the stability of the numerical calculation. In this study, the number of residual blocks was set to 12.

#### 2.2.2. The Optimization of ProbNet

For the optimization of ProbNet, the design of the training objective function was a core work. Once the training objective function was determined, a gradient descent algorithm was applied to reduce the training objective function step by step until a predefined condition was met. Hereinafter, we will detail the training objective function.

For the vanilla RANSAC algorithm, its schema was as follows: supposing there was a putative matched point set X, a minimum matched point set $X_{i}$ was drawn from the set X uniformly, then a geometric model hypothesis $h_{i}$ was calculated by using the minimum set $X_{i}$. Following the above process, we iterated *N* times, so that we finally had a pool of model hypotheses $H=(h_{1},\ldots ,h_{N})$. The final geometric transformation model $\hat{h}$ was acquired according to a measure function *s*:

(1) $$\hat{h}=\underset{h\in H}{arg max}s\left(h,X\right)$$

For ProbNet-RANSAC, the biggest difference was the method of sampling the minimum matched point set $X_{i}$ from the putative matched point set X. Instead of uniform random sampling, it sampled the minimum matched points according to a probability distribution p∼p(X;w), which was generated by ProbNet with parameters w. Here, p(X;w) denotes a categorical distribution over the putative matched points. In practice, model estimations $\left(h_{1},\ldots ,h_{N}\right)$ from H were independent of each other. Therefore, the probability of p(H;w) was defined as follows:

(2) $$p\left(H;w\right)=\prod_{i=1}^{N}p\left(h_{i};w\right),\mathrm{with}\;p\left(h_{i};w\right)=\prod_{j=1}^{M}p\left(x_{i};w\right)$$

where *M* denotes the cardinality of the sampled minimum matched point set. When the model hypotheses pool of H was acquired, the final model transformation $\hat{h}$ could be easily solved by Equation (1).

The parameters w of ProbNet would be updated in a direction that increased the probability of selecting outlier-free minimum sets, which would increase the chances of acquiring a more accurate geometric transformation model $\hat{h}$. To update the parameters w effectively, a task-loss function $\ell (\hat{h})$ that could measure the quality of the estimated model had to be designed. The commonly used task-loss function was the number of inliers of the final geometric transformation model. Therefore, the essence of ProbNet learning was to learn a distribution p(H;w) in a way that there was a high probability of receiving a small task loss. Inspired by the policy gradient approaches in reinforcement learning that would minimize a loss function defined over a stochastic process [28], the training objective function of ProbNet was designed as the minimization of the expected task loss:

(3) $$L\left(w\right)=E_{H∼p(H;w})\left[\ell (\hat{h})\right]$$

The gradient of the expected task loss with respect to the network parameters w was derived as follows:

(4) $$\frac{\partial }{\partial w}L\left(w\right)=E_{H∼p(H;w)}\left[\ell \left(\hat{h}\right)\frac{\partial }{\partial w}\log p\left(H;w\right)\right]$$

The possible hypotheses were a combinatorial problem, and the number of possible hypotheses was very large. Therefore, integrating all the possible hypothesis pools was impractical. In practice, we approximated the gradients by drawing *K* samples $H_{k}∼p\left(H;w\right)$:

(5) $$\frac{\partial }{\partial w}L\left(w\right)\approx \frac{1}{K}\sum_{k=1}^{K}\left[\ell \left(\hat{h}\right)\frac{\partial }{\partial w}\log p\left(H_{k};w\right)\right]$$

The gradient of the task-loss function *ℓ* did not appear in the above equation. This implied that the differentiation ability of the task-loss function was not required, which added great flexibility to the design of the task-loss function. Due to the random nature of sampling, the gradient variance of Equation (5) was very high. According to the variance reduction in the community of reinforcement learning [29], an average amount was subtracted from Equation (5), and we had the following expression:

(6) $$\frac{\partial }{\partial w}L\left(w\right)\approx \frac{1}{K}\sum_{k=1}^{K}\left[\left[\ell \left(\hat{h}\right)-m\right]\frac{\partial }{\partial w}\log p\left(H_{k};w\right)\right]$$

where $m=\bar{\ell }$ denotes the average loss per image.

## 3. Experiment and Results

In this section, the effectiveness and performance of the proposed image registration method were demonstrated in the qualitative and quantitative experiments, respectively. In the following, we will detail the generation of training data, the training of ProbNet, the qualitative experiment, and the quantitative experiments.

### 3.1. The Generation of Training Data

**Figure 4:**
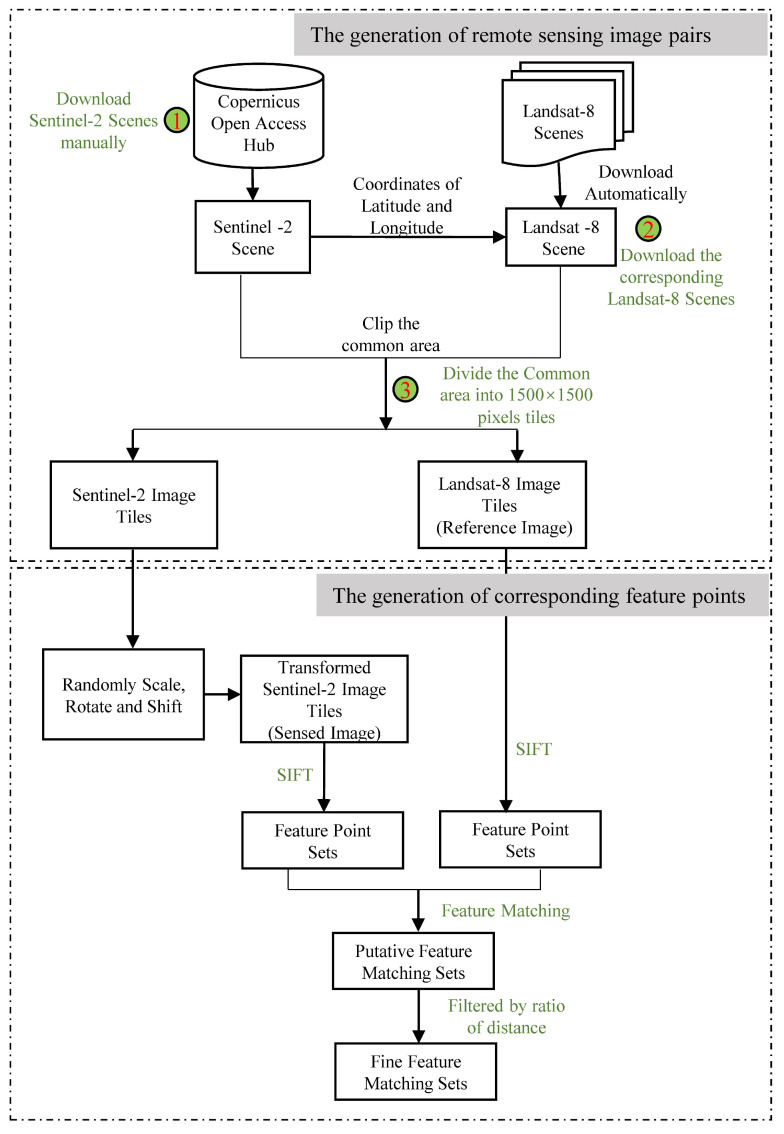
The processing flow chart of generation of training data. It consisted of two sub-processes including the generation of remote sensing image pairs and the generation of corresponding feature points. In the generation of remote sensing image pairs, we firstly downloaded Sentinel-2 scenes manually, then according to the coordinates of latitude and longitude, we downloaded the corresponding Landsat-8 scene automatically, and we finally extracted the common areas and divided them into image tiles with a size of 1500×1500 pixels. In the generation of corresponding feature points, we randomly transformed the Sentinel-2 image tiles by scaling, rotating, and shifting them to acquire the sensed image and regarded the Landsat-8 image tiles as the reference image. We used the SIFT algorithm to extract the feature points from the reference and sensed image and acquired the putative feature matching sets according to the distance of feature descriptors and further filtered out the wrong feature matches by a threshold of the ratio of nearest and second feature descriptor distances.

At the time of our study, there were no publicly available large remote sensing image registration data sets. However, the preparation of the training data was a very time-consuming and tedious work. In this study, we proposed a semi-automated method to generate training data, which reduced the workload greatly. The processing flow chart is shown in Figure 4.

To detail the processing of the generation of training data, we divided it into two steps, as follows:

1. The generation of remote sensing image pairs. The Level-1C products of Sentinel-2 and Level-2 products of Landsat-8 remote sensing images were used as image sources. Firstly, we manually downloaded the Sentinel-2 images from the Copernicus Open Access Hub. Secondly, for each Sentinel-2 image, we used the python script to automatically download the corresponding Landsat-8 image according to the coordinates of latitude and longitude of the Sentinel-2 image. Finally, we cropped the common area of the pair of images by using the GDAL [30] and divided the common area into image tiles with a size of 1500×1500 pixels. Each pair of image tiles was aligned due to the Level-1C products of Sentinel-2 and Level-2 products of Landsat-8 having been rigorously geometrically corrected.
2. The generation of corresponding feature points. For each pair of image tiles, we regarded the Landsat-8 image tile as a reference image, and then randomly scaled, rotated, and shifted the Sentinel-2 image tile to mimic the geometrical deformation between the image pairs and regarded the transformed tile as a sensed image. The value of the scaling, rotation, and displacement formed a homography matrix *H*. The range of rotation was $\left(-30^{\circ },30^{\circ }\right)$, and the range of scaling was (0.8,1.25), and the range of shift was (−100,100) pixels. After the generation of the reference and sensed image pairs, we used the SIFT algorithm from the VLFeat MATLAB library [29] to detect feature points from each image and match them to acquire the putative corresponding feature points. Then we used the ratio of the nearest-to-second-nearest descriptor distance to filter out obviously wrong correspondence points in order to acquire the fine feature corresponding sets. The threshold of ratio was set to 1.5. The fine corresponding point set for each pair of images was regarded as a sample of the training data, and its label was the homography matrix *H*. The number of corresponding feature points was different for each pair of images due to the difference between image contents.

To increase the diversity of the training data, the coverage of the scene images was varied among farmland, desert, buildings, water, mountains, and so on. Furthermore, the image acquisition time spanned all four seasons of the year, from 2018 to 2021. The cloud coverage of each image was less than 10%. The total number of image pairs were 1609, and the number of corresponding feature points for each image was different, from 50 to 3881.

### 3.2. The Details of Training of ProbNet

For the training of ProbNet, two important aspects were the design of the task-loss function and the initialization of ProbNet. The task-loss function guided how the network was optimized and determined, to a large extent, the final performance of the network. However, due to the highly non-convex nature of the loss function, the initialization of ProbNet not only affected the convergence speed of the network, but also affected whether the network could converge. In the following, we detail the loss function and the initialization of ProbNet.

In this study, three different loss functions were used to train ProbNet. They were mean error of reprojection error, negative number of inliers, and negative F1 score. Notably, in the community of image registration, for a specific geometric transformation model, one of its inliers was a pair of corresponding feature points that obeys the geometric transformation model itself. In addition, for the mean error of reprojection error *ℓ*, its formula was expressed as follows:

(7) $$\ell =\frac{1}{N^{\prime }}\sum_{i=1}^{N^{\prime }}\left(d\left(x_{i},\hat{H}x_{i}^{\prime }\right)+d\left({\hat{H}}^{-1}x_{i},x_{i}^{\prime }\right)\right)$$

where $\hat{H}$ is the estimated homography matrix, $N^{\prime }$ is the number of corresponding feature points determined by $\hat{H}$, $d\left(x,x^{\prime }\right)=\sqrt{{\left(x_{0}-x_{0}^{\prime }\right)}^{2}+{\left(x_{1}-x_{1}^{\prime }\right)}^{2}}$, and $\left(x_{i},x_{i}^{\prime }\right)$ is a corresponding feature point determined by the homography matrix $\hat{H}$. $\hat{H^{-1}}$ denotes the inverse matrix of $\hat{H}$. For the loss of a negative number of inliers, the criteria of determining a pair of corresponding feature points as inliers was the reprojection error being less than 1 pixels. For the loss of a negative F1 score, F1 was calculated as follows:

(8) $$F1=2*\frac{Precision*Recall}{Precision+Recall}$$

For the initialization of network, we optimized the KL-divergence of the sampling probability of $\hat{P}=\left(\hat{p}\left(x_{0},x_{0}^{\prime }\right),\ldots ,\hat{p}\left(x_{n},x_{n}^{\prime }\right)\right)$ predicted by ProbNet for each corresponding feature point, and a target sampling probability of $P=\left(p\left(x_{0},x_{0}^{\prime }\right),\ldots ,p\left(X_{n},x_{n}^{\prime }\right)\right)$ was calculated by the reprojection error according to the data from the homography matrix *H*. The expression for the KL-divergence was calculated as follows:

(9) $$KL(\hat{P}\left||P\right)=\sum_{i=0}^{n}\left(p\left(x_{i},x_{i}^{\prime }\right)*\log\frac{p(x_{i},x_{i}^{\prime })}{\hat{p}\left(x_{i},x_{i}^{\prime }\right)}\right)$$

where *n* is the number of corresponding feature points to the sample data, and the target sampling probability of each corresponding feature point was a function of its reprojection distance to the ground accuracy of the homography matrix, its expression was given in Equation (10) in the section of discussion about the initialization of ProbNet. The learning rate was set to $5\times 10^{-4}$ and decayed it by 0.95 every 5 epochs. The total number of initialization epochs was 3000. The other hyper parameters were set as follows: the number of iteration of RANSAC, *N* in Equation (2), was set to 5; the cardinality of the sampled minimum matched point set, *M* in Equation (2), was set to 4; and the number of samples to approximate the gradients, *K* in Equation (5), was set to 6.

After completing the initialization of ProbNet, we trained the network of ProbNet by minimizing its task-loss function using Adam [31] with an initial learning rate of $1\times 10^{-3}$ and decaying the learning rate every 5 epochs. The decaying rate was 0.95. Due to the different number of corresponding feature points for each sample, the batch size could only be set to 1. The total number of training epochs was 300. Other hyper parameters, such as the number of iterations of RANSAC, the cardinality of the sampled minimum matched point set, and the number of samples to approximate the gradients had the same value as when we initialized of ProbNet. Our method was implemented in Pytorch. Both our training and initialization was performed on two RTX TITAN 24 G GPUs.

### 3.3. Qualitative Experiment

**Figure 5:**
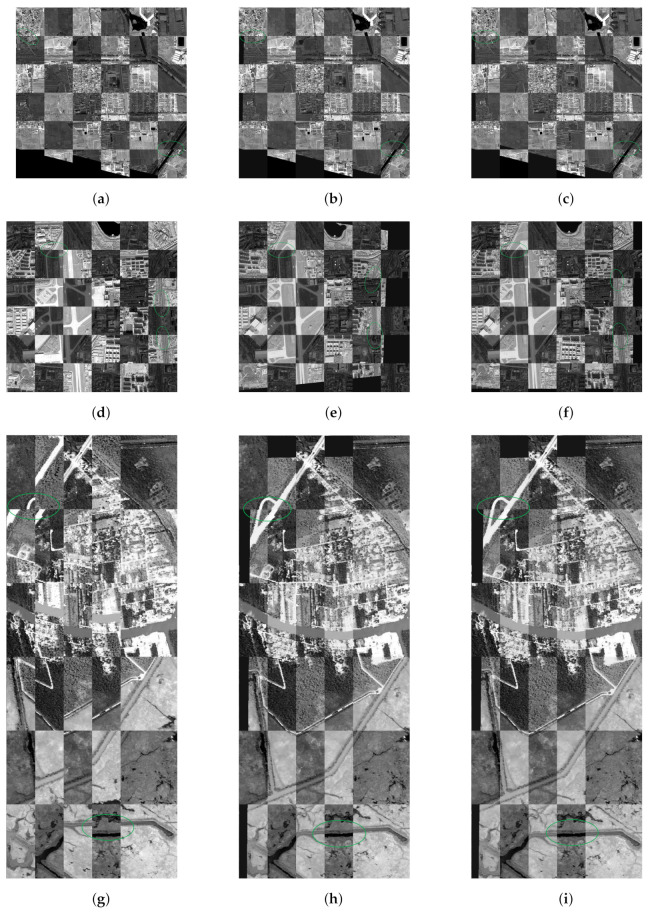
Checkerboard visualization for the qualitative experiments. The first column of images (**a**,**d**,**g**), the second column of images (**b**,**e**,**h**) and the third column of images (**c**,**f**,**i**) are the checkerboard visualizations of the original unregistered images, images registered by vanilla RANSAC, and images registered by ProbNet-RANSAC, respectively. Some areas circled by green ellipses contain distinctive ground features, from which it was easy to determine the result of image registration. Take each row of images as a unit, each circled area of one image has one corresponding circled area in the other two images; they almost cover the same content. For the circled areas in the unregistered images, their ground features were faulted, and it was hard to recognize them as a whole, which showed the geometric deformations between images. For the circled areas in the images registered by the method of vanilla RANSAC, their ground features were not strictly aligned, while for the circled areas in the images registered by the method of ProbNet-RANSAC, their ground features were aligned.

To illustrate the effectiveness of the network of ProbNet, two pairs of GF-1 (Gaofen-1) and GF-2 (Gaofen-2) images and one pair of hyperspectral images were applied to the register. GF-1 and GF-2 belonged to the series of high-resolution optical Earth observation satellites of the China National Space Administration. The experimental GF-1 image was a panchromatic band with a resolution of 2 m and a size of 1024×1024 pixels. The experimental GF-2 image was also a panchromatic band, but its resolution was 0.8 m and its size was 2500×2500 pixels. The experimental hyperspectral images [32], (named as Crown Point 2010 and Crown Point 2011) had a size of 540×1400, and their resolutions were 3.5 m and 3.4 m, respectively. The scenes these three pairs of images covered were different: the first one contained an airport, the second contained farmland, a lake, and buildings, and the last contained human-made buildings, a river, forests, and roads. In the schema of RANSAC, we sampled the minimum set of corresponding feature points 50 times under the guidance of the probability of each corresponding feature points that had been generated by the trained network of ProbNet, and finally we obtained the optimal homography transformation matrix *H* according to the measurement of the re-projection error. For a more visual display of the registration results, the checkerboard visualization method was adopted. Due to the large scale difference, the GF-2 panchromatic image was down-sampled to the same resolution as the GF-1 panchromatic image. The checkerboard visualization of the original and registered images is shown in Figure 5. Salient features with fault displacement in the original images are circled with green ellipses, and the corresponding salient aligned features are also circled by the green ellipses in the images registered by ProbNet.

### 3.4. Quantitative Experiments

**Figure 6:**
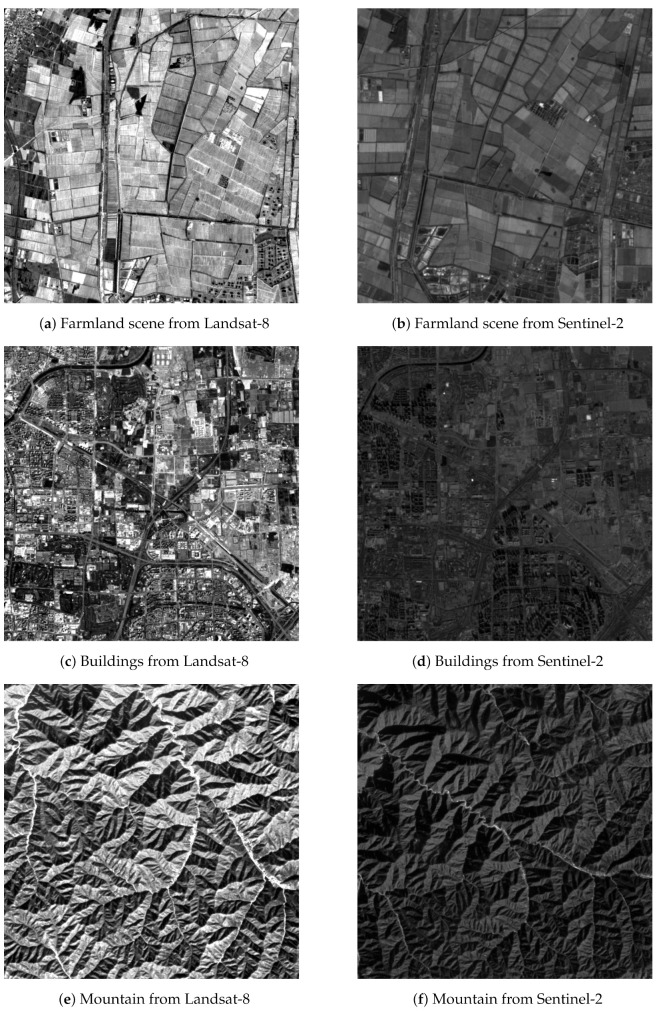
Three examples of test image pairs. (**a**,**b**) were one pair of images; they covered a scene of farmland. (**c**,**d**) were one pair of images; they contained human-made buildings, such as buildings, roads, and so on. (**e**,**f**) were one pair of images; they covered a mountain area. The images in the left column were from Landsat-8 panchromatic bands, and the images in the right column were from Sentinel-2 bands. The geometric distortion of each pair of images included the differences of scale, rotation, and displacements.

To evaluate the performance of the proposed method quantitatively, three other representative methods were introduced to compare. The first one was a vanilla RANSAC method [25], which treats all corresponding feature points equally and samples the corresponding feature points uniformly; the second one was ProSAC [21], which draws the minimum sets from progressively larger sets of the top-ranked correspondences; and the last one was LMeds-RANSAC, which minimizes the least median of squared error distance. The vanilla RANSAC was implemented by our team, and the ProSAC and LMeds-RANSAC were implemented in OpenCV Library [33]. For the proposed method of ProbNet-RANSAC, we trained it by using the different loss functions. One was ProbNet-RANSAC_Inlier, which was trained by the loss function of the negative number of inliers; the other was ProbNet-RANSAC_F1, which was trained by the loss function of a negative F1 score. A total of 200 pairs of Landsat-8 and Sentinel-2 images were used as test images. The test image pairs were generated in the same way as the training data. The geometric deformation for each pair of images was random, the angle of image rotation was in the range of $\left(-30^{\circ },30^{\circ }\right)$, the displacement between images was in the range of (−100,100) pixels, and the scale between images was in the range of (0.8,1.25). The scenes covered by the test images were varied from farmland, human-made buildings, rivers, deserts, mountains, and so on. In addition to the different scenes, the imaging times and seasons were different, so the images had large gray differences or even some image contents were changed. Three examples from these test images are shown in Figure 6.

**Figure 7:**
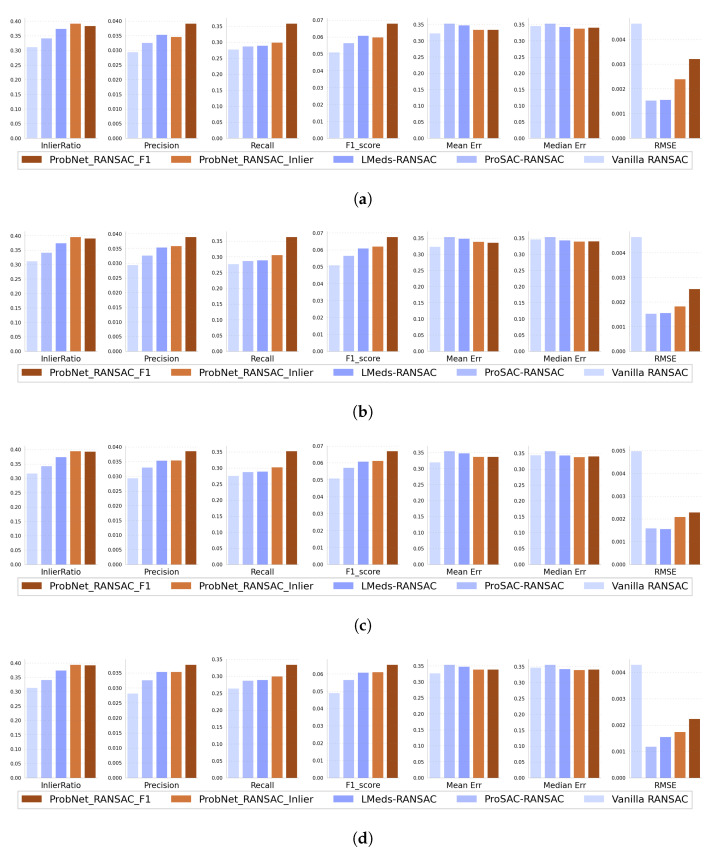
The bar graph of InlierRatio, Precision, Recall, F1_score, Mean Error, Median Error, and RMSE of different methods at 50, 500, 1000 and 2000 iterations. (**a**) The bar graph of different measures of different methods after 50 iterations; (**b**) The bar graph of different measures of different methods after 500 iterations; (**c**) The bar graph of different measures of different methods after 1000 iterations; (**d**) The bar graph of different measures of different methods after 2000 iterations.

For each method, seven measures of InlierRatio, Precision, Recall, F1_score, Mean Error, Median Error, and Root-Mean-Square Error (RMSE) were averaged to compare their performance. For the sake of presentation, some variables for each image pair were defined as follows: *N* was the total number of correspondence feature points, *H* was the ground accuracy of the transformation, $\hat{H}$ was the estimated transformation, $N_{1}$ was the number of inliers determined by the estimated transformation of $\hat{H}$, and $N_{2}$ was the number of inliers determined by the ground accuracy transformation *H*. For InlierRatio, it was the ratio of $N_{1}$ to *N*, for Precision, Recall and F1_score, as they were the commonly used measures in classification and object recognition methods and were calculated according to a confusion matrix. Mean Error, Median Error, and RMSE were calculated for the estimated inliers. The experimental results are illustrated in the following Figure 7.

## 4. Discussion

In this section, the effectiveness of the proposed method of ProbNet-RANSAC are analyzed and the performance of the proposed method are discussed.

### 4.1. Analysis of Qualitative Experiment

By comparing the checkerboard visualization of the original images and the images registered by ProbNet-RANSAC in Figure 5, we found that some salient linear features were strictly aligned visually, and the registration result was satisfactory. This showed that the effectiveness of the proposed method. Furthermore, the iteration of RANSAC was only 50, while vanilla RANSAC could not achieve the same comparable results in the case of only 50 iterations, according to the registration results of vanilla RANSAC and comparing the corresponding circled areas in the images registered by vanilla RANSAC and ProbNet-RANSAC. Due to the large gray difference between images and some changes of content, some corresponding feature points detected were contaminated by noises; some were outliers. This showed that the probability generated by the network of ProbNet played a vital role in guiding the sampling of the minimum point set in the schema of RANSAC and was beneficial to improve the performance of image registration. In addition, the sensors of GF-1 ang GF-2 images were different from the ones of the training images of Sentinel-1 and Landsat-8; this also indicated the generalizability of the network of ProbNet to other optical remote sensing images.

### 4.2. Analysis of Quantitative Experiment

In the quantitative experiment, a total of seven measures were used to evaluate the performance of each method. They were roughly divided into two groups. The first group included four measurements: InlierRatio, Precision, Recall, and F1_score; the larger they were, the better the performance of the method. The second group included the three remaining measures: Mean Error, Median Error, and RMSE; the smaller they were, the better the performance of the method. These two groups of measures evaluated the performance of the method from different perspectives and were somewhat complementary. The Mean error, the Median error, and the RMSE of each method were calculated for the inliers, as determined by their respective estimated transformation models.

In terms of measures in the first group, namely InlierRatio, Precision, Recall and F1_score, both ProbNet-RANSAC_Inlier and ProbNet-RANSAC_F1 had superior performance, or at least slightly superior performance, as compared to the other three methods. Specifically, as compared to vanilla RANSAC, the proposed method had superior performance by a large margin. Combining the only difference in sampling the minimum samples from putative corresponding feature sets, namely vanilla RANSAC sampled uniformly while ProbNet-RANSAC sampled by the probability distribution generated by ProbNet-RANSAC; therefore, we could conclude that the probability generated by ProbNet played a vital role and could effectively guide sampling to improve the performance of the image registration. As compared to the other two methods, ProSAC-RANSAC and LMeds-RANSAC, the proposed method also outperformed them. In terms of some measures in the second group, namely Mean Error and Median Error, the proposed methods had slightly lower or almost equal values, as compared to the other three methods, while for the measure of RMSE, the proposed method had a lower value than vanilla RANSAC, but had a higher value than the other two methods, ProSAC-RANSAC and LMeds-RANSAC. The reason for the performance advantage of the proposed methods was that these measures were only calculated for their respective inliers for each method. However, according to the measure of InlierRatio, the number of inliers for each method was different; the proposed methods had the largest number of inliers, and vanilla RANSAC had the least. The smaller number of inliers may have been biased towards the lower value of the Mean and Median Errors. This is further discussed in the effects of loss function of ProbNet.

In terms of the number of iterations, we estimated the performance of each method in different iterations of 50, 500, 1000, and 2000. Interestingly, in the process of increasing the number of iterations from 50 to 2000, the performance of each method was not significantly improved, except by a significant reduction of RMSE of the proposed methods. This showed that simply increasing the number of iterations could not improve the performance of the method. For further analysis, we took a pair of images with 800 corresponding feature points as an example. There were $C_{800}^{4}$ different minimum sets with 4 corresponding feature points in total, while 50, even 2000 minimum sets, accounted for a very small percentage. Therefore, the chance of acquiring a better transformation by increasing the iterations was very small, and the contribution made by increasing the iteration to further improve the performance of the method was insignificant. At the same time, this also demonstrated the importance of obtaining high-quality minimum sets of points under the guidance of probability, as generated by ProbNet.

### 4.3. The Effects of Loss Function of ProbNet

Theoretically, any task-loss function could be used to train ProbNet, as the differentiation ability of the loss function was not required, as illustrated by Equation (5). Therefore, for the task of image registration based on feature, we adopted three different loss functions, namely reprojection error, negative number of inliers, and negative F1 score. For ProbNet-RRANSAC_Inlier trained by minimizing the negative number of inliers and ProbNet-RRANSAC_Inlier trained by minimizing the negative F1 scores, their performances are shown in Figure 7. In terms of Recall, Precision, and F1 score, the performance of ProbNet-RANSAC_F1 was superior to that of ProbNet-RANSAC_Inlier. However, in terms of InlierRatio, Mean Error, and Median Error, their performance was almost equal, while for the RMSE, the performance of ProbNet-RANSAC_F1 was inferior to ProbNet-RANSAC_Inlier. Therefore, it was hard to distinguish which of them was better. However, for the reprojection loss during training, a failure occurred: As the reprojection error decreased gradually, the number of inliers also decreased. This showed the bias of the loss-of-reprojection error. If we had pursued the minimization of the projection error, it would have led to a significant bias in the transformation estimation. This also showed that the single reprojection error did not fully assess the estimated transformation, so the number of inliers must be taken into account. Moreover, this was consistent with the phenomenon that the proposed methods had a slight performance advantage over the other three methods in terms of the measures in the second group.

### 4.4. The Initialization of ProbNet

**Figure 8:**
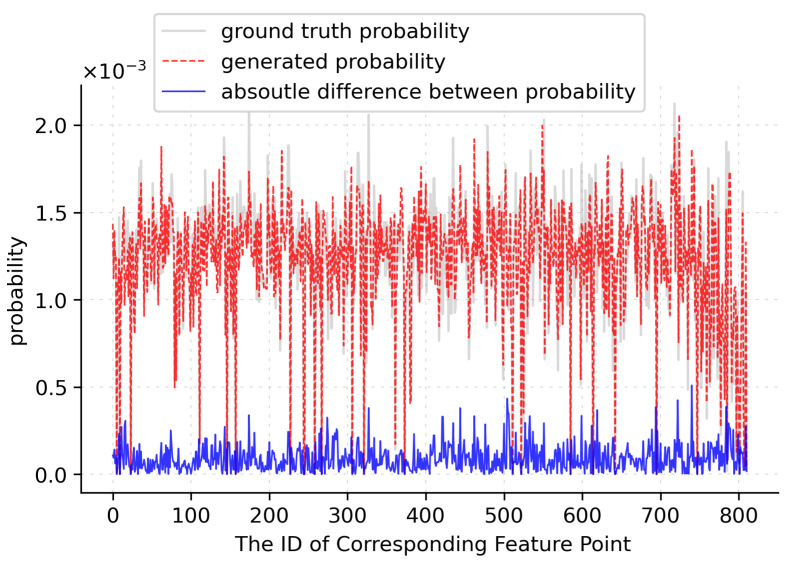
The comparison of generated probability distribution and ground accuracy probability distribution. The gray curve denotes the ground accuracy probability, the red dashed curve denotes the generated probability, and the blue curve denotes the absolute difference between generated probability and ground accuracy probability.

Due to the loss function being a non-convex function, the optimization was sensitive to the initialization of ProbNet. To accelerate the training of ProbNet, we initialize it by minimizing the Kullback–Leibler divergence between the probability distribution $\hat{p}\left(x;\omega \right)$ generated by ProbNet and the ground accuracy probability distribution p(x;E). The ground accuracy probability for each corresponding feature point was defined as the following expression:

(10) $$p\left(x,E\right)=M\frac{1}{\sqrt{2\pi }\sigma }\exp\left(-\frac{d(x,E)}{2\sigma^{2}}\right)$$

where σ is the threshold to determine whether one pair of corresponding feature points is an inlier, and d(x,E) denotes the reprojection error of the corresponding feature with respect to the ground accuracy of geometrical transformation, and *M* is the normalizer, which is used to normalize the sum of the probability of all the corresponding features for a pair of images, to 1. We initialized for 3000 epochs using Adam with a learning rate of $1\times 10^{-4}$. The comparison between the probability distribution generated by ProbNet and the ground accuracy probability distribution for one example image is shown in Figure 8.

As shown in the figure, the probability distribution generated by ProbNet was very similar to the ground accuracy probability distribution, and the absolute probability difference of each corresponding feature point was also very small, much lower than the average probability of $1.23\times 10^{-3}$. The smaller the σ, the narrower the shape of the ground accuracy probability, which indicated that fewer corresponding feature points had large probabilities. To increase the generalization of ProbNet, the σ was set to a relatively large value of 1.2 in our experiment.

### 4.5. The Efficiency of ProbNet Ransac

For the proposed method of ProbNet-RANSAC, the main modification was using ProbNet to generate the probability of each corresponding point. To measure the additional time-consuming step, we compared it with vanilla RANSAC, which assigns the equal probability of each corresponding point directly. The experimental images were the 200 pairs of images in the quantitative experiment. The iterations were equal to 2000. The average elapsed times of a pair of images for vanilla RANSAC and ProbNet-RANSAC were 1.1549 s and 1.1925 s, respectively. As we expected, the elapsed times of ProbNet-RANSAC were larger than those of the vanilla RANSAC. However, for the methods of ProSAC-RANSAC and LMeds-RANSAC, their average elapsed times of a pair of images were in the order of thousandths of a second, much lower than those of vanilla RANSAC and ProbNet-RANSAC. This was due to ProSAC-RANSAC and LMeds-RANSAC being implemented by OpenCV, where their code was fully optimized. Whereas Vanilla RANSAC and ProbNet-RANSAC were implemented manually using Python. Considering that the overall time required for an image registration included the elapsed time of feature extraction and image resampling, the increased elapsed time was acceptable.

## 5. Conclusions

Due to large gray differences, noises, cloud occlusions, image content changes, and complex geometric deformations between remote sensing image pairs from different sensors or taken at different times, there are always noises and false matching points in the putative feature corresponding point set acquired in the process of image registration based on feature. Furthermore, the number of the false matching points or matching points with noises is always high, which degrades the accuracy of image registration and can lead to image registration failures. In this study, we built a deep convolutional neural network of ProbNet to predict the sampling probability of each corresponding feature point in the putative corresponding feature point set. Then the predicted probability was used to guide the sampling of the minimum set of RANSAC to acquire a more accurate transformation model estimation in order to improve the accuracy of image registration. Through qualitative and quantitative experiments, as well as a detailed discussion and analysis, we draw the following conclusions:

- The proposed method could effectively register different remote sensing images; its registration result was satisfactory by the checkerboard visualization of images after registration, and it should be generalizable to other optical remote sensing images.
- Regarding the three different task losses including reprojection error, the negative F1 score, and the negative number of inliers, the minimized reprojection error would bias ProbNet towards a smaller number of inliers, while a negative number of inliers or a negative F1 score could effectively optimize ProbNet.
- To accelerate the training of ProbNet, a special initialization of ProbNet was conducted by minimizing the Kullback-–Leibler divergence between the ground accuracy probability distribution and predicted probability distribution for each corresponding feature point. After 3000 epochs, the predicted probability was a good approximation to the ground accuracy probability.
- Regarding the measures of InlierRatio, Precision, Recall, and F1 score, the proposed methods trained by minimizing the negative number of inliers or negative F1 score had significant advantages over the other three popular methods including vanilla RANSAC, ProSAC RANSAC, and LMeds RANSAC. However, for the measures of Mean Error, Median Error, and RMSE, the advantages of the proposed method diminished due to these measures being calculated for their respective inliers; the number of inliers of the proposed methods were the largest. This was also supported by the experiment of minimizing the task loss of reprojection error, and the smaller number of inliers also led to a decrease in the reprojection error.

Overall, the probability generated by ProbNet plays a vital role in the schema of RANSAC, and the proposed method of ProbNet-RANSAC_Inlier and ProbNet_F1 had superior performance over other methods, including vanilla RANSAC, ProSAC RANSAC and LMeds RANSAC. This also showed the potential of deep convolutional neural networks in the field of image registration. By integrating the deep convolutional neural network of feature extraction [16] and feature descriptor [34] with ProbNet, a deep convolutional neural network could be developed in future research, and it could be trained by fully end-to-end to jointly optimize each process of image registration based on feature in order to further improve the accuracy of image registration. In addition, ProbNet-RANSAC has the potential to improve the accuracy of medical image registration based on feature by fine-tuning the training data of medical images.
